# Reducing Antibiotic Use in a Level III and Two Level II Neonatal Intensive Care Units Targeting Prescribing Practices for Both Early and Late-onset Sepsis: A Quality Improvement Project

**DOI:** 10.1097/pq9.0000000000000555

**Published:** 2022-06-14

**Authors:** Doron J. Kahn, Beckett S. Perkins, Claire E. Barrette, Robert Godin

**Affiliations:** From the * Division of Neonatology, Joe DiMaggio Children’s Hospital, Hollywood, FL; † Mednax Services, Inc., Sunrise, FL; ‡ Department of Pharmacy, Joe DiMaggio Children’s Hospital, Hollywood, FL.

**Keywords:** antibiotic management, antibiotic guidelines, antibiotic stewardship program, neonatal sepsis, antibiotic utilization rate

## Abstract

**Introduction:**

Variation in antibiotic (ATB) use exists between neonatal intensive care units (NICUs) without demonstrated benefit to outcomes tested. Studies show that early-onset sepsis occurs in up to 2% of NICU patients, yet antibiotics (ABX) were started in over 50% of neonates admitted to our NICUs. An internal audit identified variations in prescribing practices and excessive use of ABX. As a result, we introduced ATB stewardship to our NICUs in 2015 to reduce unnecessary usage of these medications.

**Methods:**

We used standard quality improvement methodology utilizing multiple iterative plan-do-study-act cycles during a 6-year project to test various interventions aimed at using ABX wisely. Specifically, our goals were to reduce ABX on admission (AA), percent of patients who continued on ABX beyond 72 hours of life (AC), and ATB utilization rate in our 3 NICUs by 28% for each metric. Interventions implemented included the development of an ATB stewardship program consisting of a multidisciplinary team that met regularly, creation of tools and guidelines for evaluations of sepsis and ATB use, universal use of the neonatal early-onset sepsis calculator for all newborns 34 weeks and older gestational age, education regarding noninitiation of ABX for maternal indications in clinically well newborns, and discontinuation within 48 hours for asymptomatic newborns with negative blood cultures.

**Results:**

AA, AC, and ATB utilization rate decreased by 34.1%, 45.3%, and 34.9%, respectively, in our 3 NICUs.

**Conclusions:**

By introducing ATB stewardship in our NICUs, we exceeded our predetermined goal of significantly reducing ATB usage.

## INTRODUCTION

It is well known that inappropriate antibiotic (ATB) use increases morbidity and mortality in neonates.^1,2^ Misuse of antibiotics (ABX) exposes patients to risks of adverse effects and contributes significantly to the growing problem of ATB resistance.^1–3^ ATB exposure in the first days of life alters normal microflora colonization important to intestinal health.^4^ Short and long-term implications of this exposure include associations with increased rates of obesity,^5^ asthma, eczema and allergy,^6^ inflammatory bowel disease,^7^ diarrhea,^8^ nephrotoxicity,^9^ ototoxicity,^10^ sepsis including fungal infections,^11^ necrotizing enterocolitis (NEC),^8,12^ bronchopulmonary dysplasia,^13,14^ retinopathy of prematurity,^14^ neurodevelopmental impairment,^15^ and death.^1,12–15^

According to the Centers for Disease Control and Prevention (CDC), 20%−50% of all ABX prescribed in hospitals in the United States are deemed unnecessary or inappropriate.^16^ Variation in ATB use exists between neonatal intensive care units (NICUs) without demonstrated benefit.^17^ Hospital-based ATB stewardship program (ASP) interventions decrease ATB use, reduce antimicrobial resistance, avoid potential medication errors and associated adverse events, and save healthcare dollars.^18,19^ ABX given beyond 48 hours of life in neonates without culture-proven sepsis is particularly wasteful.^20^ National and international organizations have recently pushed to support the introduction of ASPs into healthcare systems.^21,22^ The Vermont Oxford Network (VON) instituted ATB stewardship in a multicenter fashion via a quality improvement (QI) collaborative.^21^ Through choosing ABX Wisely based on the 7 core elements of ATB stewardship from the CDC,^23^ VON demonstrated a significant and clinically important reduction in ATB utilization rate (AUR).^20^ These initiatives are having a positive effect, as noted by Magill et al,^24^ who documented a downward trend in ATB usage among 148 hospitals over 5 years.

Like other units at the time, we identified variation in ATB prescribing practices and excessive use of ABX in our NICUs. As a result, we introduced ATB stewardship in 2015 to reduce unnecessary use of these medications, specifically targeting ABX on admission, prolonged ATB use in neonates with negative admission blood cultures, and AUR.

## METHODS

### Setting

Memorial Healthcare System (MHS) comprises 5 hospitals, 3 of which have maternity services and NICUs. The largest of these, Joe DiMaggio Children’s Hospital (JDCH), is an 84-bed NICU with 62 level III beds and 22 level II beds in a regional tertiary referral center. The other 2 are level II NICUs; Memorial Hospital West (MHW) has 33 beds, and Memorial Hospital Miramar (MHM) has 16 beds. JDCH averages nearly 5,000 deliveries/y, and each level II hospital has approximately 4,000 deliveries/y. Approximately 10% of admissions to JDCH are outborn. The same neonatology group (15 neonatologists and 21 advanced practice practitioners [APPs]) who worked for Envision Healthcare Services (EHCS) at the time of this project and are contracted employees with the healthcare system staff all 3 NICUs. All 3 NICUs share the same policies, including automatic admission criteria for babies <35 weeks gestational age (GA) or <2000 g birth weight, and a sepsis evaluation guideline that incorporates the use of the neonatal early-onset sepsis (EOS) calculator (available at: https://neonatalsepsiscalculator.kaiserpermanente.org) for all newborns 34 weeks and older GA. For more details on the NICUs’ settings, see **Supplemental Digital Content 1,** http://links.lww.com/PQ9/A372.

### Subjects

We included all neonates admitted to an MHS NICU in the QI project.

### Study Design and Program Description

**Fig. 1.**
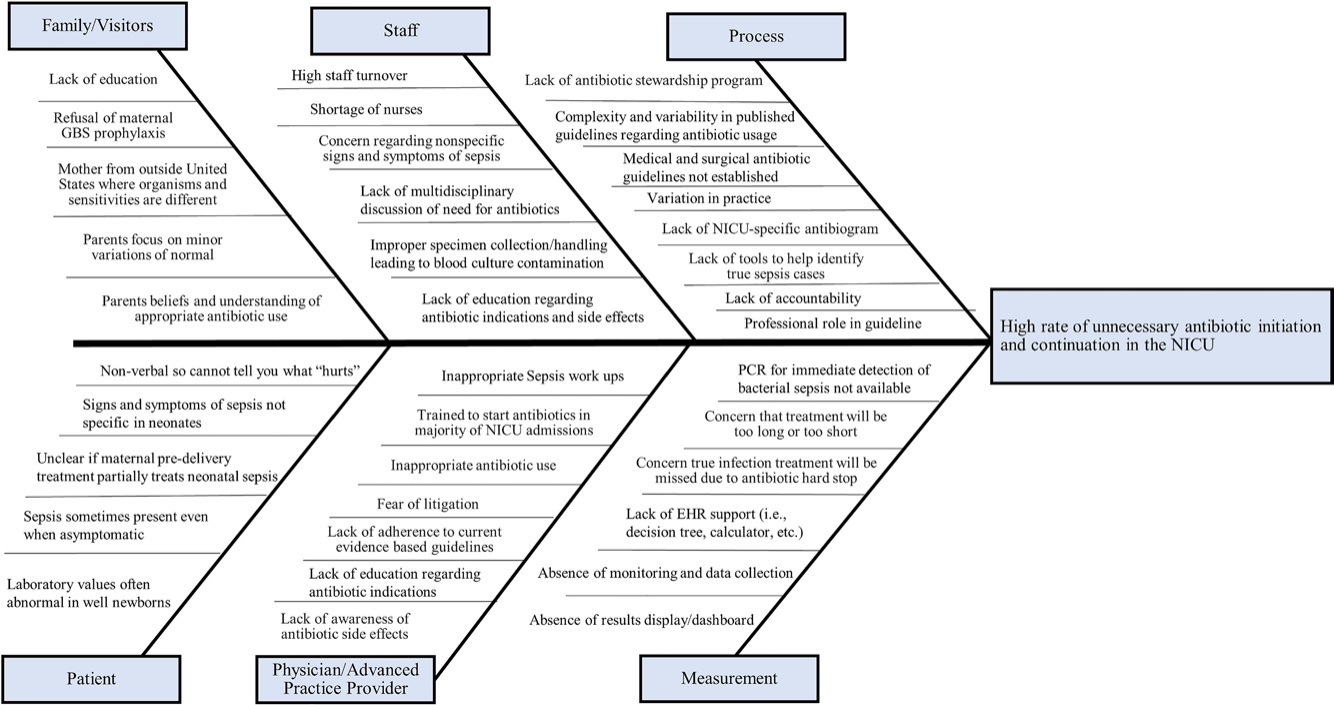
Antibiotic stewardship Ishikawa cause-and effect (“fishbone”) diagram.

**Fig. 2.**
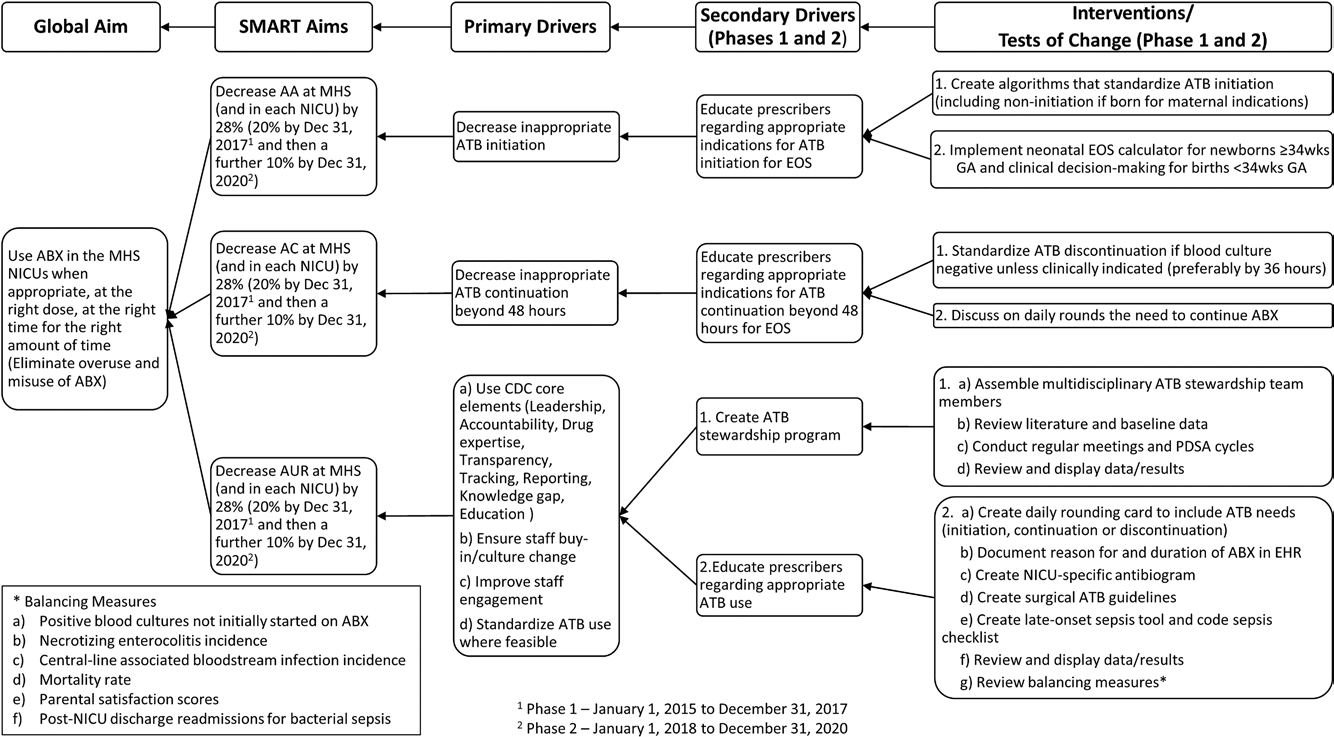
Antibiotic stewardship key driver diagram. Numbers 1 and 2 refer to phase 1 (January 1, 2015, to December 31, 2017) and phase 2 (January 1, 2018, to December 31, 2020), respectively. AA, antibiotics on admission; ABX, antibiotics; AC, antibiotics continued beyond 72 hours; ATB, antibiotic; AUR, antibiotic utilization rate; CDC, Centers for Disease Control and Prevention; EHR, electronic health record; EOS, early-onset sepsis; GA, gestational age; MHS, Memorial Healthcare System; NICU, neonatal intensive care unit; PDSA, plan-do-study-act; SMART, specific, measurable, achievable, relevant, time-bound.

In 2014, the authors realized a need to introduce ATB stewardship into the MHS NICUs based on an internal survey demonstrating variation in ATB prescribing practices among members of the neonatology group. We presented various clinical scenarios to all 14 neonatologists and 16 APPs in our group. The survey results demonstrated little consistency in ATB prescribing practices (see **Supplemental Digital Content 2,** http://links.lww.com/PQ9/A372). Team members for ATB stewardship first met in December 2014, and we launched the MHS NICUs ASP on January 1, 2015. The initial team was composed of the NICU medical director, a NICU quality specialist, a neonatal pharmacy clinical specialist, frontline registered nurses, and an ex-NICU parent (1 of our APPs). In 2017, an infectious disease physician, infection control specialist, another neonatal pharmacy clinical specialist, and a pediatric surgeon joined the team. We collected baseline data from January 1, 2014, to December 31, 2014. We used available evidence in the literature before January 1, 2015, to create a QI project with QI methodology utilizing multiple iterative plan-do-study-act cycles to use ABX wisely in our NICUs (see **Supplemental Digital Content 3,** http://links.lww.com/PQ9/A372). Our committee met every 4−8 weeks to plan, enact, and study each intervention, then decided whether to adopt or abandon the intervention, and the cycle would repeat. We revised and updated the project as new evidence emerged, especially in 2016 with the release of VON’s Internet-based Neonatal Improvement Collaborative for Quality (iNICQ) “Choosing Antibiotics Wisely” potentially best practice toolkit.^25^ We created an Ishikawa cause-and-effect (“fishbone”) diagram and a driver diagram with specific, measurable, achievable, realistic, and time-bound (SMART) aims and drivers (see Figs. 1 and 2, respectively). These diagrams evolved as elements were added and removed from the project based on adoption or abandonment during the plan-do-study-act cycles. We focused on 4 key CDC potentially best practice elements of ATB stewardship: (1) ensuring an organizational commitment to ATB stewardship; (2) developing, testing, implementing, and refining policies appropriate for ATB stewardship; (3) applying pharmacy-driven interventions to ensure appropriate ATB use, and (4) reporting regularly to the staff.^20^ We presented results at separate quarterly meetings to the neonatology group, NICU staff, hospital-wide ASP committee, and as abstracts in poster format every other year at the annual VON quality congress.

Various ATB stewardship metrics have been reported in the literature. We chose to study 3 that we thought would maximally reduce unnecessary ATB usage. (1) The percent of patients started on ABX on admission (AA) is a frequent target of ASPs given this metric’s variability across NICUs and due to the disproportionality of true sepsis cases compared with the number of newborns started on ABX.^26^ (2) The percent of patients continued on ABX ≥ 72 hours of life (AC) is another metric that has considerable potential to reduce unnecessary ATB usage due to the common scenario of continuing their use in babies with abnormal symptoms or laboratory values but without positive blood cultures.^26^ (3) AUR, defined as the number of patient days infants receive ABX per the number of patient days in the NICU, is a standard measure of ATB usage reported in the literature and used in databases compared with other hospitals.^17,20^ EHCS provides neonatology services to over 40 NICUs across the nation and collects the 3 metrics listed earlier from these hospitals for comparison. All 3 hospitals at MHS performed worse than the means for EHCS hospitals on all 3 metrics in 2014. We defined initial SMART aims to reduce AA, AC, and AUR by 20%, in line with both internal company best quartile aims and what was reported as the best quartile in the literature.^17^ In January 2018, we set new goals of reducing AA, AC, and AUR by an additional 10% each over the subsequent 2 years through December 2019 (yielding a total reduction goal of 28% to align ourselves with goals set in the VON iNICQ collaboration)^25^ and sustain these improvements in 2020. We tracked data quarterly to provide adequate power to determine true differences over time and assess the various interventions’ effects. In addition to standard ATB stewardship metrics, we calculated the number of neonates on admission and the number of NICU days spared ABX over the 6-year project to provide more concrete improvement figures to the NICU staff. These values were calculated based on annual data compared with baseline data (January 1, 2014, to December 31, 2014) and adjusted for the number of admissions and number of NICU days in subsequent years.

We performed random chart audits to confirm adherence to newly established guidelines throughout the project. Also, to ensure the effectiveness of change and that no adverse effects were caused by changes in policies/recommendations (balancing measures), we performed case reviews of all infants with bacterial sepsis not evaluated or treated within 2 hours of birth, and those discharged from the NICU and readmitted within 30 days of life with bacterial sepsis. We also monitored infections (including central line-associated bacterial infections [CLABSIs]), central line days, NEC rates, mortality, and parental satisfaction (using Press Ganey scores) as part of routine quality and safety data collection.

The MHS Institutional Review Board determined this study is exempt from the requirement for Institutional Review Board oversight. The authors declare no conflict of interest. For more details on the NICUs’ study design and program description, see **Supplemental Digital Content 1,** http://links.lww.com/PQ9/A372.

### SMART Aims

Our SMART aims were to reduce AA, AC, and AUR within MHS by 28% step-wise over 5 years from January 1, 2015, to December 31, 2019, compared with January 1, 2014, to December 31, 2014, and sustain improvement throughout 2020. Specifically, we aimed to reduce AA from 57.5% to 41.4%, AC from 18.1% to 13%, and AUR from 21.5% to 15.5%.

Subgroup analyses were performed on each of the 3 hospital’s data within MHS (JDCH, MHW, and MHM) and also stratified by GA weeks (≤31, 32−33, 34−36, and ≥37) using these same goals of reducing AA, AC, and AUR by 28%. These GA cutoffs were chosen as neonates born 31 weeks and younger are often started on ABX due to nonspecific clinical symptomatology despite no risk factors for sepsis, 32−33 week GA births are often observed in our NICUs without starting ABX if clinically well, the neonatal EOS calculator can be used for births 34 weeks and older, and 37 weeks and older GA differentiates term from preterm births.

## RESULTS

Table 1 summarizes the major interventions that drove changes in ATB prescribing practices in our NICUs. Random chart audits confirmed adherence to newly established guidelines, including using the neonatal EOS calculator for newborns 34 weeks and older GA and ABX deferred for maternal conditions in the absence of clinical symptomatology and compliance with surgical ATB guidelines in 100% of cases.

**Table 1.** Major Antibiotic Stewardship Interventions

1. Created a multidisciplinary neonatal intensive care unit-specific ATB stewardship team composed of a neonatologist, infectious disease physician, infection control specialist, quality specialist, pharmacist, APP, frontline nursing and respiratory staff, and nursing leadership.
2. Educated on and standardized use of the Centers for Disease Control and Prevention algorithm refined with recommendations from the Committee on Fetus and Newborn from 2010 to 2014 in conjunction with use of the neonatal early-onset sepsis calculator for every birth 34 weeks and older GA. This latter step reassured neonatologists/APPs and pediatricians that sepsis workups and ABX are often not indicated, and in the majority of cases it is safe to observe asymptomatic newborns without starting ABX.
3. Established culture of noninitiation of ABX for babies delivered for maternal indications (ie, pregnancy-induced hypertension, intrauterine growth restriction), if clinically behaving like their GA. Excluded premature infants younger than 34 weeks GA on more than minimal respiratory support who will require ATB initiation and continuation until blood cultures are negative.
4. Changed from “Counting doses” to “Complete X days of treatment” to avoid administering extra doses of ABX when dose or medication changes.
5. Encouraged discontinuation of ABX *before* 48 hours for early-onset sepsis. Every 12 hour Ampicillin (last dose at 36 hours) and every 24 hour Gentamicin (last dose at 24 hours) will protect baby through 48 hours.
6. Encouraged ATB discontinuation when blood culture negative at 36−48 hours for late-onset sepsis.
7. Created standardized pre- and postoperative order set in the electronic health record for surgical conditions.
8. Recommended 5-day ATB treatment course for diagnosed pneumonia.
9. Created neonatal antibiogram to assist in making educated empiric ATB choices.
10. Created late-onset sepsis tool and code sepsis checklist^27^ to assist in making multidisciplinary decisions regarding need for sepsis workups and ABX.

ABX, antibiotics; APP, advanced practice practitioner; ATB, antibiotic; GA, gestational age.

**Fig. 3.**
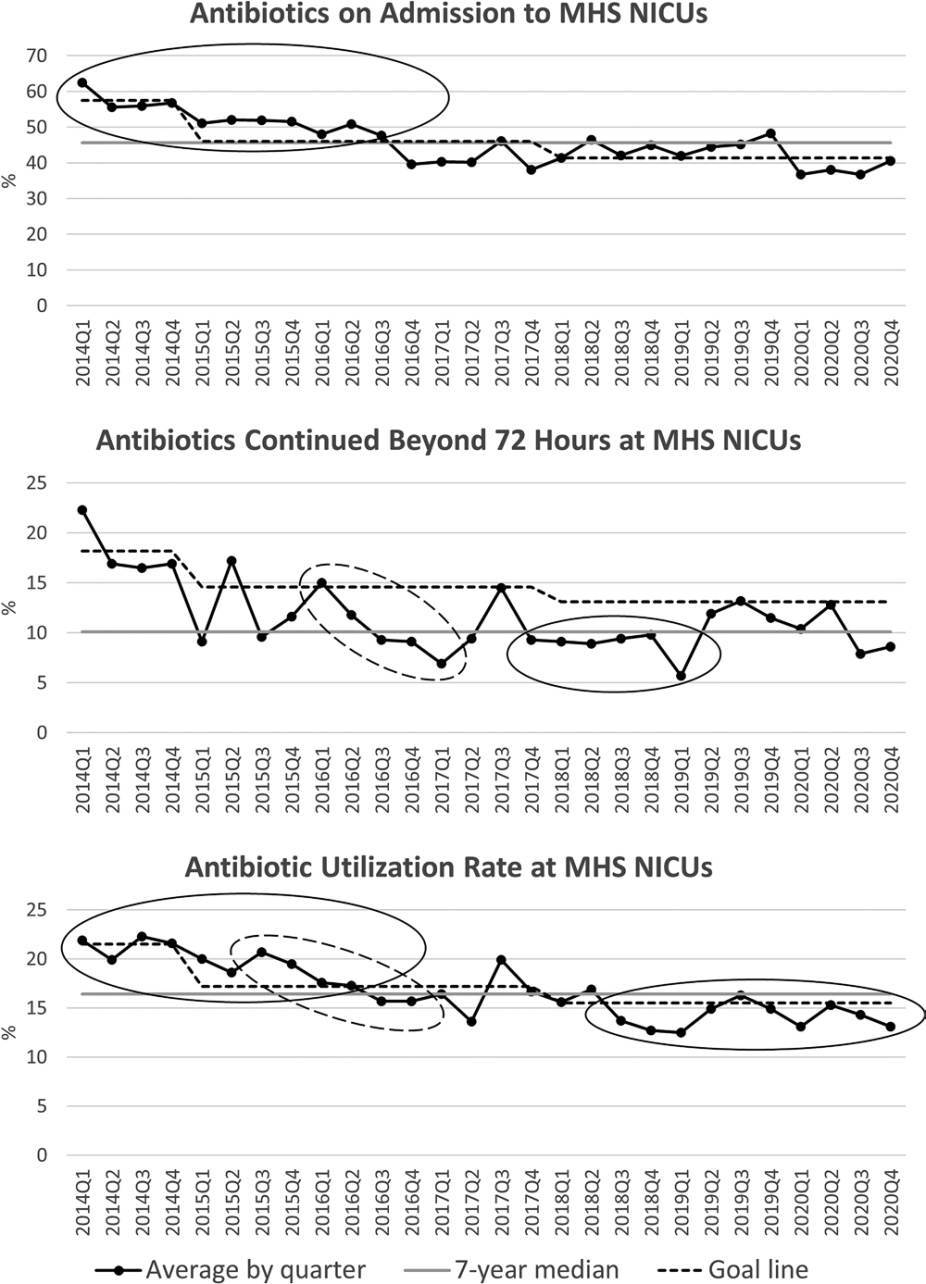
Antibiotic stewardship run charts. Goal-line set as baseline period mean for first 4 quarters (2014), 20% reduction from baseline period for subsequent 3 years (2015−2017), and an additional 10% reduction for final 3 years (2018−2020). Seven-year median based on all years of the project, including baseline period. Solid and dashed ovals represent shifts (at least 6 points above or below the median) and trends (at least 5 points in the same direction). Run charts show improvements in both shifts and trends for all 3 metrics throughout the 7-year project. MHS, Memorial Healthcare System; NICUs, neonatal intensive care units.

Run charts for each of the 3 metrics within the entire hospital system (Fig. 3) and each hospital and gestational age group individually (see **Supplemental Digital Contents 4 and 5**, http://links.lww.com/PQ9/A372) demonstrate a steady reduction in all 3 measures over the 6-year project. In most run charts, successful shifts, trends, or both demonstrated improvement in targeted metrics. For 5 quarters in the hospital-specific data, there were adverse trends (increasing AA at JDCH and MHW); however, no adverse trends were noted for any of the primary analyses or other subgroup analyses (see **Supplemental Digital Content 4,** http://links.lww.com/PQ9/A372). Sustainability data (2020) compared with baseline data (2014) shows that we reached our goals of significant reductions in ATB usage, both trending over time and an absolute reduction exceeding the predetermined goals (see **Table 3, Supplemental Digital Content 6**, http://links.lww.com/PQ9/A372). GA-specific data indicates that we successfully reduced ATB usage for all 3 metrics in all GA categories. Greater reductions in ATB usage were demonstrated for those neonates born 32−36 weeks GA for AA, ≤33 weeks GA for AC, and 34 weeks and older GA for AUR (see **Table 4, Supplemental Digital Content 6**, http://links.lww.com/PQ9/A372). From 2015 to 2020 over 1,000 patients were spared ABX on admission, and over 10,000 patient-days were spared ABX compared with baseline (see **Tables 5 and 6, Supplemental Digital Content 6**, http://links.lww.com/PQ9/A372).

Balancing measures were not impacted during the study period. Cases of delayed initiation of ABX (newborns who were started on ABX after 2 hours of life) occurred in 14 out of 47 cases of EOS (29.8%), but upon review would have occurred regardless of which algorithm or guideline we used as a majority of these newborns were initially asymptomatic with minimal to no maternal risk factors for EOS (see **Table 7, Supplemental Digital Content 6**, http://links.lww.com/PQ9/A372). The mean (SD) delay in ATB initiation in these 14 newborns was 12.6 (7.6) hours, and only 1 newborn had ABX delayed more than 24 hours. All 14 newborns whose ABX were delayed were discharged home without known complications after 10 to 14 days of ABX. NICU mortality and NEC rates remained stable, and CLABSI rates decreased over the 6 years of the project (data not shown). Thirty-day readmission rates decreased, and those due to invasive bacterial infections (bacteremia or meningitis) were not different during the 5 years before project initiation compared with the 6-year duration of this project (see **Table 8, Supplemental Digital Content 6**, http://links.lww.com/PQ9/A372). Upon individual case reviews, we found no readmissions for bacterial sepsis due to alterations in our practice of managing bacterial sepsis in the NICU.

## DISCUSSION

In 2014, the neonatal community focused on reducing unnecessary ATB use based on papers from the American Academy of Pediatrics Committee on Fetus and Newborn, enhancing and clarifying the 2010 CDC algorithm on ATB prophylaxis for maternal Group B Streptococcus sepsis.^28–31^ Unfortunately, the recommendations lacked details to address all clinical scenarios. An internal survey of our neonatology group found that we were not practicing consistently for various clinical vignettes. Other institutions have reported similar variations in the evaluation and treatment of EOS.^32^ Despite studies showing EOS occurs in approximately 0.05% of newborns overall,^33^ and up to 2% of NICU admissions,^32^ we started ABX in >50% of neonates admitted to our NICUs. This practice prompted us to standardize our ATB prescribing practices and reduce unnecessary use of these medications.

By creating an ASP and introducing measures to use ABX appropriately, we realized a significant and consistent reduction in ATB use in our NICUs. Upon subgroup analysis, only 2 adverse trends were noted (increasing AA for 5 quarters at 2 of the individual hospitals). These 2 adverse trends may represent normal variability due to a greater percentage of births with significant maternal risk factors for sepsis or clinically symptomatic births during that time. In contrast, all of the primary analyses and most of the subgroup analyses demonstrated improved shifts or trends. Our level II NICUs and level III NICU showed similar improvements, indicating that ASP can be applied successfully and equally among units. GA-specific data indicates improvements for all metrics and across all GA categories; however, some GA groups performed better. Prescribers found it easier to withhold ABX at birth in 32−33 and 34−36 week GA newborns, likely due to the former being asymptomatic and the latter having low neonatal EOS calculator scores. Only a modest reduction in ATB prescribing at birth was noted for newborns 31 weeks and younger GA as they usually present with at least mild respiratory symptoms, which may or may not be due to sepsis. Similarly, reduction of AA for term newborns was less robust than those 32−36 weeks GA as newborns 37 weeks and older GA are often admitted to the NICU due to clinical symptomatology. All GA categories demonstrated significant improvements in avoiding prolonged ABX for EOS, although, as once blood cultures result as negative, prescribers are comfortable discontinuing ABX.

### Limitations

Implementation of a NICU-specific ASP, although challenging from a time, labor-intensive, and emotional perspective (given the strong opinions surrounding ATB usage in the NICU), is feasible and can improve prescribing practices for these medications. However, significant barriers to the development and implementation of this project included time outside of clinical responsibilities (to create the program, attend meetings, educate personnel, audit charts, and analyze and present data), drafting key players, encouraging involvement, and overcoming preset opinions regarding the need for ABX in certain clinical scenarios (Table 2).

**Table 2.** Antibiotic Stewardship Challenges and Mitigation Strategies

Challenge	Mitigation Strategy
Overcoming meeting logistics (room availability and space especially during the Covid-19 pandemic)	Help administration to understand importance of an ASP to garner support with logistics
Procuring time apart from clinical responsibilities	Arrange meetings just before or just after shift change for staff convenience
Creating a dedicated ASP team	Present importance of ASP
Adjusting to preset practices	Present literature support, show data and balancing measures, and be open about cases of delayed inititation of ABX (missed cases of sepsis)
Eliminating practice variation	Create standardized guidelines
Securing EHR support	Use EHR to ease burden of data collection
Ensuring time for data analysis and presentation	Identify committed staff members accountable for data analysis and synthesis and development of presentations
Disseminating information	Present data at quarterly meetings, display posters throughout the unit, maintain updated dashboards, and keep staff and stakeholders informed through frequent emails
Educating regarding new processes	Educate through formal sessions explaining new guidelines, and informal 1-on-1 sessions based on need
Garnering feedback/satisfaction	Encourage reporting of delayed/missed sepsis workups for discussion, and survey staff
Monitoring compliance	Review charts, using automated EHR wherever possible
Ensuring ABX are discussed on daily rounds	Add discussion of ABX to hand-off tools (nursing I-PASS and neonatologist/advanced practice practitioner sign-out sheet), and make part of the unit culture

ABX, antibiotics; ASP, antibiotic stewardship program; EHR, electronic health record; I-PASS, illness severity, patient summary, action list, situational awareness and contingency planning, synthesis by receiver.

Other challenges included meeting time and space, electronic health record changes/upgrades, frequent need for process improvement cycles, communicating these changes to sizeable staff, 3 different NICUs with competing priorities, and nursing leadership in transition. Given these various NICU-specific and personnel issues, education of new initiatives was often unsystematic, occurring at random times and with whichever ASP team members and staff were available. We did not garner ASP team-member, staff, and parental satisfaction in a formalized fashion but rather through casual queries, although the feedback was universally positive. Also, given the breadth of a project involving multiple interventions, we did not monitor compliance with the various change interventions (ie, neonatal EOS calculator use rate, electronic health record documentation of reason for starting/continuing ABX, ATB discussion rate on daily rounds) in a formalized way, but rather through random chart and rounding audits.

We recognized fear of failure to identify bacterial sepsis in neonates who might only present with risk factors or subtle symptoms. Sharing reviews of all bacterial sepsis, NEC, CLABSI, and mortality cases, and all readmissions for sepsis, demonstrated to the neonatology group that we were able to realize significant reductions in ATB usage without sacrificing balancing measures. This result is supported in the literature as summarized in a meta-analysis by Achten et al, which found a relative risk of empiric ATB use of 0.45 (confidence interval 0.35−0.57) with equivalent rates of missed sepsis (28% versus 29%) using the neonatal EOS calculator compared with conventional management by an algorithm.^34^ Reassuringly, our incidence of delayed ATB initiation (29.8%) was similar to what is reported in the literature (28%−59%)^34,35^ despite our “delayed” definition of 2 hours compared with 24 hours in the literature. Also, sharing evidence with the neonatology group that approximately two-thirds of EOS cases are either asymptomatic at birth and/or with few maternal risk factors^35^ allayed fears that inappropriate delays in ABX initiation were occurring or that delays were due to failure of the neonatal EOS calculator and/or guideline. Many of the above interventions and challenges are not unique to our NICUs. The fact that we successfully implemented this program in both small and large units suggests that they can be applied to other units challenged with overprescribing ABX.

## CONCLUSIONS

We created a NICU-specific ATB Stewardship team, then implemented a rigorous education process for the NICU team and general pediatricians, all of whom supported an evidence-based reduction in ATB use in our neonates. As a result, we realized a significant reduction in ATB usage, both trending over time and an absolute reduction exceeding our predetermined goals. In addition to presenting a success story of safe ATB stewardship implementation into our NICUs, we set a new paradigm by providing a model to effect similar initiatives in other units.

## ACKNOWLEDGMENTS

The authors acknowledge the assistance of the MHS NICU neonatologists, advanced practice practitioners, nurses, pharmacy clinical specialists, infectious disease specialists, and our infection control specialist.

## DISCLOSURE

The authors have no financial interest to declare in relation to the content of this article.

## Supplementary Material


