# Supplementary material for: Reducing Antibiotic Use in a Level III and Two Level II Neonatal Intensive Care Units Targeting Prescribing Practices for Both Early and Late-onset Sepsis: A Quality Improvement Project
Source: Pediatr Qual Saf. 2022 Jun 14;7(3):e555. doi: 10.1097/pq9.0000000000000555 (PMC9197376; doi:10.1097/pq9.0000000000000555)
Supplement: Supplementary file 1 [file pqs-7-e555-s001.pdf]

Supplemental Digital Content 1

Supplemental Methods and Material:

Setting

Memorial Healthcare System (MHS) comprises five hospitals, three of which have maternity services and neonatal intensive care units (NICUs). The largest of these, Joe DiMaggio Children's Hospital (JDCH), is an 84-bed NICU with 62 level III beds and 22 level II beds in a regional tertiary referral center. Forty-three of these beds are single patient rooms (which can accommodate twins) and have rooming-in capability for the parents, and the other 41 beds are located within four open pods. The other two NICUs are level II NICUs; Memorial Hospital West (MHW) has 33 beds, and Memorial Hospital Miramar (MHM) has 16 beds. JDCH averages nearly 5000 deliveries/year, and each level II hospital has approximately 4000 deliveries/year. Approximately 10% of admissions to JDCH are outborn of whom a majority are transferred from our two level II NICUs. In all three NICUs, we perform routine nursing interventions (i.e., bathing, weighing, heel sticks), minor surgical procedures (i.e., lumbar punctures, chest tube insertions), and emergency surgical operations (i.e., peritoneal drain placement for spontaneous intestinal perforation) at the bedside. Most major planned surgeries are performed in the JDCH operating rooms. The same neonatology group, who worked for Envision Healthcare Services (EHCS) at the time of this project and are contracted employees with the healthcare system, staff all three NICUs. The group is comprised of 15 neonatologists and 21 advanced practice providers (APPs). The neonatologists and APPs all rotate through the three NICUs so all members of the group are exposed to, manage, and prescribe antibiotics (ABX) to neonates at all three hospitals. Some of the nurses (RNs) provide shift coverage at multiple hospitals, but most remain staffed at one of the three hospitals. Intravenous access is never initiated in the newborn nurseries (NBNs) of the three hospitals, therefore all babies requiring intravenous ABX are admitted to one of the three NICUs. Approximately 80% of the babies in the NBN are managed by any of a number of pediatric groups and private pediatricians, and the neonatology group manages the remaining 20% whose parents have not yet selected a pediatrician or the pediatrician does not have privileges at MHS, and those in whom a

pediatrician has requested a consultation. In addition, all three NICUs share the same policies including automatic admission criteria for babies less than 35 weeks GA and/or less than 2000g birth weight, and a sepsis evaluation guideline which incorporates the use of the neonatal early-onset sepsis calculator (Available at: https://neonatalsepsiscalculator.kaiserpermanente.org) for all newborns ≥34 weeks gestational age GA. The neonatology group establishes policies in the NBN, including those related to sepsis screening, however private pediatricians have autonomy to manage newborns while following established guidelines, or place a neonatology consultation (which may lead to admissions to the NICU for possible intravenous ABX).

Study design and program description

The primary objectives during the first phase of the QI initiative (2015-2017) were to 1) establish a reliable and sustainable NICU ASP including key members from each NICU discipline; 2) collect baseline data before project initiation; 3) review the literature for potentially best practices and educate on and implement these best practices (i.e., withhold ABX from newborns delivered for maternal indications, discontinue ABX before 48 hours if indicated by negative blood culture(s), begin use of the neonatal EOS calculator for births ≥ 34 weeks GA), and 4) present data to staff.

The primary objectives during the PDSA cycles in the second phase of this QI initiative were to establish a new unit culture focused on ATB stewardship by 1) continuing to educate and guide staff regarding unnecessary ATB use (i.e., We added prompts “Does the baby need ABX?” and “Are ABX still needed and for how long?” to the nursing bedside I-PASS [illness severity, patient summary, action list, situational awareness and contingency planning, synthesis by receiver] communication tool to ensure ABX were discussed on daily rounds for all babies); 2) standardizing initiation of ABX based on the use of the neonatal EOS calculator for every birth ≥ 34 weeks GA; 3) encouraging decision-making regarding ATB initiation based on sepsis risk factors and clinical presentation for births <34 weeks GA; 4) encouraging ATB discontinuation before 48 hours when blood culture remains negative and low clinical suspicion for sepsis; 5) using standardized pre- and post-op order sets in the EHR for every neonate with a surgical condition; 6) normalizing use of an internally-created late-onset sepsis (LOS) tool and code

sepsis checklist,[27] and 7) continuing to report results to all NICU staff. Data were collected on every ATB encounter via MHS programming through our EHR (Epic) and checked for accuracy through random audits.

We followed standard QI methodology using multiple PDSA cycles to test various interventions to use ABX wisely and eliminate unnecessary ATB usage in the NICU. Behavior changes among prescribers and staff members were instrumental in driving process improvements. We held regular meetings involving a multidisciplinary team of NICU providers, which maintained interest and buy-in that reducing ATB use was not only supported by evidence in the literature but was also safe and beneficial for both short and long-term outcomes. Prescribers changed their ATB prescribing practice from automatically starting ABX on admission unless a strong reason not to existed (pre-project inception), to being suspect that the majority of NICU admissions do not need ABX despite the ASP team's recommendations (phase 1), and finally to understanding that ABX are not warranted in the majority of cases (phase 2 and sustainability phase). Having support from ID physicians and an infection control specialist reassured providers that experts in the field supported the new ATB stewardship recommendations. Pharmacists assisted in optimizing ATB dosing by clarifying prescribing endpoints and updating EHR order sets. Front line staff reminded team members to discuss the need for ABX on daily rounds. NICU staff (neonatologists, APPs, pharmacists, and frontline workers) progressed from believing "our patients are in the NICU and therefore are sick and so ABX are likely needed" (pre-project) to "prescribers are focused on ordering less ABX, and we will follow along" (phase 1), and finally to "ABX are not safe for our babies when not clinically indicated so we must work as a team to evaluate the need for ABX in every baby in the NICU daily" (phase 2). Over time, discussions of ATB needs became as routine on rounds as discussions of respiratory and nutritional needs. Pharmacists attending rounds ensured ABX (along with other clinically relevant medications) were discussed daily. Frontline nursing staff also progressed from passive recipients of ATB orders to "healthy microbiome advocates" for their patients, often initiating the discussion of whether ABX were still needed. Seeing real-time data and celebrating milestones (i.e., 1,000 babies spared ABX on admission or 10,000 days spared ABX since project inception) were motivating factors that helped maintain interest in the project. We reviewed the literature frequently and made changes to recommendations throughout the project based on the latest evidence. There was a shift from an algorithm-based guideline to

the neonatal EOS calculator, then ultimately to a stronger reliance on clinical symptomatology that yielded lower ATB usage rates over time. Also, we created a subcommittee from the larger group to focus on more accurate identification and timely treatment of LOS cases.

Global Aim

We designed our QI project to standardize the use of ABX in the MHS NICUs to use these medications safely and smartly, starting with administering the right drug, at the right dose, using the proper concentration, with the proper route, the proper infusion period, at the right time, and for the right length of time, to improve patient outcome, reduce the risk of harm, and optimize family and provider satisfaction.

Supplemental Digital Content 2

Supplemental Table 1. Variation in management of hypothetical clinical rule-out sepsis vignettes

9 Question survey[a]

How do you manage the following scenarios? (The first 4 refer to a GBS negative mother who did not receive intrapartum ABX and has no risk factors for sepsis other than those listed):

1. CS, ROM at delivery, 35 weeks 1 day – 36 weeks 6 days?
2. NSVD, ROM ≤18 hours, 35 weeks 1 day – 36 weeks 6 days?
3. NSVD, ROM >18 hours, 35 weeks 1 day – 36 weeks 6 days?
4. NSVD, ROM >18 hours, ≥37 weeks 0 days?
5. GBS prophylaxis indicated in mom, inadequately treated, and either <37 weeks *or* ROM >18 hours: follow CDC or COFN?
6. GBS prophylaxis indicated in mom, inadequately treated, and <37 weeks *and* ROM ≤18 hours: follow CDC or COFN?
7. GBS prophylaxis indicated in mom, inadequately treated, and ≥37 weeks *and* ROM >18 hours: follow CDC or COFN?
8. Persistently abnormal lab data in asymptomatic newborn?
9. Screening labs at 12 hours to routinely include CRP?

Results[b]

In agreement (>85%)

1. Late preterm, CS – do not screen (89%)
3. Late preterm, NSVD and ROM >18 hours – screen (89%)
9. Do not do routine CRPs (86%)

Somewhat in agreement (65-85%)

5. GBS prophylaxis indicated in mom, inadequately treated, and either <37 weeks *or* ROM >18 hours – screen with CBC/blood culture [CDC] (preferred over CBC alone [COFN]) (82%)
6. GBS prophylaxis indicated in mom, inadequately treated, and <37 weeks *and* ROM ≤18 hours – screen [CDC] (preferred over observation [COFN]) (75%)
7. GBS prophylaxis indicated in mom, inadequately treated and ≥37 weeks *and* ROM >18 hours – do not screen, observe [COFN] (preferred over screen [CDC]) (72%)

Most divisive (<65%)

2. Late preterm, NSVD, ROM ≤18 hours – do not screen (64%)
4. Term, NSVD, ROM >18 hours – screen (64%)
8. Persistently abnormal lab data in asymptomatic newborn – do not admit/do not treat (57%)

[a] Survey distributed to 14 neonatologists and 16 advanced practice providers within the Memorial Healthcare System (with a 94% response rate) in November 2014 during the planning stages of our antibiotic stewardship program.

[b] % refers to the majority % of providers who answered the same for a particular survey question

CBC, complete blood count; CDC, Centers for Disease Control and Prevention; COFN, Committee on Fetus and Newborn; CRP, c-reactive protein; CS, caesarean section; GBS, Group B Streptococcus; NSVD, normal spontaneous vaginal delivery; ROM, rupture of membranes.

Supplemental Digital Content 3

Supplemental Table 2. Plan do study act (PDSA) Cycles and tests of change

**PDSA cycles**	**Interventions/Tests of change**
**PDSA 1: Sep 2014**<br><br>Established neonatal intensive care unit (NICU) Antibiotic Stewardship Program (ASP) team	*Plan:* Discussed need for best practice ASP and creation of a program for implementation.<br>*Do:* Created initial team, and rolled out Envision Healthcare Services (EHCS) company-wide ASP.<br>*Study:* Discussed in general terms with all neonatology groups within EHCS. Presented monthly three metrics in the SMART Aims section of driver diagram for all 38 NICUs within EHCS, and each NICU was ranked 1 thru 38 for each metric.<br>*Act:* Established team leader and formed core group of NICU-specific ASP team members (mostly neonatologists and advanced practice providers [APPs]) to represent Memorial Healthcare System (MHS).
**PDSA 2: Oct 2014**<br><br>Introduced ASP to MHS NICUs	*Plan:* MHS NICU-specific ASP team leader presented program. Discussed antibiotic (ATB) stewardship with the MHS neonatology team in group meeting and made initial recommendations for reducing ATB usage based on Centers for Disease Control and Prevention (CDC) guideline, Committee on Fetus and Newborn (COFN) recommendations and neonatal early-onset sepsis (EOS) calculator.<br>*Do:* Educated regarding use of neonatal EOS calculator.<br>Study: Continued quarterly evaluation of data.<br>*Act:* Disseminated information to stakeholders (EHCS, neonatology group).
**PDSA 3: Nov 2014**<br><br>Educated MHS regarding ASP	*Plan:* Planned implementation for MHS NICU-specific ASP.<br>*Do:* Neonatologist team leader gave pediatric grand rounds at MHS on management of EOS (attended by neonatologists/APPs and pediatricians and all those who will be taking care of newborns).<br>*Study:* Followed compliance through antibiotic utilization rates (AURs).<br>*Act:* Educated through grand rounds, and sent summary letter to pediatricians explaining rationale for reduced sepsis workups and reduced ATB usage.
**PDSA 4: Dec 2014**<br><br>Collected baseline data	*Plan:* Identified measures.<br>*Do:* Collected baseline data 1/1/14-12/31/14.<br>*Study:* Reviewed baseline data according to company and national standards, and identified opportunities for improvement.<br>*Act:* Discussed guidelines needed.

**PDSA 5: Jan 2015**<br><br>Implemented ASP best-practice guidelines	*Plan:* Developed guidelines.<br>*Do:* Began implementation of best practice guidelines (used CDC algorithm adjusted with COFN recommendations, neonatal EOS calculator for reassurance and in cases where CDC algorithm is undefined, established 48-hours of antibiotics [ABX] for rule out sepsis, etc.), and presented quarterly data to neonatology group.<br>*Study:* Evaluated effectiveness of ASP with ongoing data collection.<br>*Act:* Adopted guidelines to be use system-wide.
**PDSA 6: 2016**<br><br>Reviewed and presented preliminary data	*Plan:* Reviewed ongoing ASP data.<br>*Do:* Continued presentation of quarterly data to neonatology group.<br>*Study:* Reviewed ongoing data from PDSA 5.<br>*Act:* Renewed commitment and focus to ASP.
**PDSA 7: Oct 2017**<br><br>Presented data at national meeting	*Plan:* ASP team attended annual Vermont Oxford Network (VON) conference.<br>*Do:* Attended VON ASP lectures and poster presentations, and discussed new ways of advancing project.<br>*Study:* VON posters and abstract reviewed identifying additional team members (registered nurses [RNs], respiratory therapists [RTs], infectious disease [ID] physicians, infection control specialist, and pharmacists).<br>*Act:* Convened team (see PDSA 8) to reassess aims.
**PDSA 8:Nov 2017**<br><br>Re-configured NICU ASP Team	*Plan:* Discussed and identified additional team members (an additional ID physician, microbiology personnel, an additional pharmacist, RN leadership, additional bedside RNs).<br>*Do:* Arranged meeting and invited multidisciplinary team to assist in continuing improvement (discussed drivers of change and interventions that will be needed, assigned members of the group to various interventions).<br>*Study:* Appraised current practices and data.<br>*Act:* Established meeting schedule. Discussed need for family advisor on team, however had difficulty identifying a family representative.
**PDSA 9: Nov 2017**<br><br>Created NICU-specific antibiogram	*Plan:* Discussed need for a NICU-specific antibiogram for ASP.<br>*Do:* Created NICU-specific antibiogram (created by ID team member and microbiology lab) so we now no longer need to rely on pediatric or adult/maternal hospital data for empiric ATB coverage.<br>*Study:* Evaluated NICU-specific antibiogram to ensure appropriate EOS antibiotic choice.<br>*Act:* Made antibiogram available for team use.

**PDSA 10: Nov 2017**<br><br>Implemented more ASP best-practice guidelines	*Plan:* Work group developed and disseminated education of current ATB stewardship measures with on-going cycles of change.<br>*Do:* Team members presented additional recommendations to neonatology team (i.e., stop ABX before the 48 hours dose, limit pneumonia treatment to five days).<br>*Study:* Realized significant culture change would be required.<br>*Act:* Developed education and tools to engage team.
**PDSA 11: Dec 2017**<br><br>Collaborated with another hospital on ASP initiatives	*Plan:* Met with multidisciplinary team from Winnie Palmer, a center for excellence, to review best practice guidelines.<br>*Do:* Team discussed and reviewed driver diagram for opportunities to reduce infection rates and practice changes to decrease septic work ups w/ blood cultures.<br>*Study:* Analyzed central line days and necrotizing enterocolitis rates.<br>*Act:* Educated neonatology team that culture and practice changes regarding ATB use have not led to increased infection rates.
**PDSA 12: Jan 2018**<br><br>Discussed ASP project update with hospital-wide ASP committee	*Plan:* Prepared project update.<br>*Do:* MHS NICU-specific ASP team leader presented project update to hospital-wide ASP.<br>*Study:* Reviewed data.<br>*Act:* Accepted feedback for process improvements.
**PDSA 13: Jan 2018**<br><br>Presented project update to neonatology group and set future goals	*Plan:* Reviewed metrics and adjusted targets after reaching four-year goals.<br>*Do:* Presented data to neonatology group and group agreed to lower goals.<br>*Study:* Studied metrics with new goal lines<br>*Act:* Goal lines adjusted on run charts.
**PDSA 14: 1/1/15-present**<br><br>Presented quarterly ASP data to neonatology group	*Plan:* ASP team reviewed best practices.<br>*Do:* Refined best practices for implementation.<br>*Study:* Continued quarterly review of data with neonatology group.<br>*Act:* Worked toward achievement and maintenance of SMART aim goals.
**PDSA 15: Mar 2018**<br><br>Standardized additional ASP-related practices	*Plan:* During team meeting and discussion realized neonatologists and APPs utilize various ways of "counting" doses.<br>*Do:* Standardized ATB usage by counting days, not doses. Bedside RNs educated regarding goal of ABX in by 60 minutes from ATB order. Daily rounding card created to ensure ATB usage gets discussed on a daily basis.<br>*Study:* Reviewed measures and data on run charts, and reviewed ABX stopped <72 hours.<br>*Act:* Created automated ATB dashboard that included original metrics as well as ABX administered by 60 minutes.

**PDSA 16: Apr 2018**<br><br>Collaborated with pharmacists for standardization of ABX ordering	*Plan:* Team identified opportunities for improvement with collaboration with pharmacy.<br>*Do:* Developed formulary restrictions and order set criteria. Changed intravenous ampicillin dosing from 100 mg/kg every 8 hours to every 12 hours.<br>*Study:* Tracked ATB metrics.<br>*Act:* Finalized pharmacy formulary chages in electronic health record (EHR) order set.
**PDSA 17: Apr 2018**<br><br>Standardized surgical ATB ordering	*Plan:* The ASP team worked with the pediatric surgery department lead for opportunities to reduce ABX for surgical patients.<br>*Do:* Created surgical pre-operative recommendations to ensure ABX were administered within one hour of surgery.<br>*Study:* Audited EHR to identify if new order-set being used.<br>*Act:* Educated nursing staff on how to release pre-operative orders.
**PDSA 18: May 2018**<br><br>Presented data at national meeting	*Plan:* Prepared VON abstract.<br>*Do:* Presented VON abstract to neonatology group showing continued improvement and opportunities for project advancement.<br>*Study:* Reviewed VON abstract run charts with neonatology group.<br>*Act:* Identified opportunities for further reduction in ATB usage, specifically not starting ABX for late preterm hypoglycemia/hypothermia.
**PDSA 19: Feb 2020**<br><br>Introduced EOS calculator system-wide	*Plan:* Introduced neonatal EOS calculator system-wide to be performed by newborn nursery and NICU RNs on all newborns.<br>*Do:* Educated all RNs system-wide.<br>*Study:* Reviewed EOS results in EHR.<br>Act: Continued to answer RN queries regarding EOS calculator use.
**PDSA 20: 1/1/20 to present**<br><br>Created and implemented LOS tool and code sepsis checklist	*Plan:* Created late-onset sepsis (LOS) tool and code sepsis checklist to aid in earlier detection of true sepsis and timely administration of ABX.<br>*Do:* Created LOS tool and code sepsis forms, educated RNs, APP and neonatologists regarding the use of these tools. Re-educated regarding timing for initiation of ABX.<br>*Study:* Reviewed time to initiation of ABX on ATB dashboard.<br>*Act:* Educated team regarding initiation of ABX within 60 minutes of order.

APP, advanced practice provider; ASP, antibiotic stewardship program; ABX, antibiotics; ATB, antibiotic; AUR, antibiotic utilization rate; EHCS, Envision Healthcare Services; EHR, electronic health record; EOS, early-onset sepsis; NICU, neonatal intensive care unit; CS, caesarean section; NSVD, normal spontaneous vaginal delivery; LOS, late-onset sepsis; CDC, Centers for Disease Control; COFN, Committee on Fetus and Newborn; MHS, Memorial

Healthcare System; PDSA, plan, do, study, act; SMART, specific, measurable, achievable, relevant, time-bound; VON, Vermont Oxford Network.

Supplemental Digital Content 4

Supplemental Figure 1. Antibiotics on admission by hospital

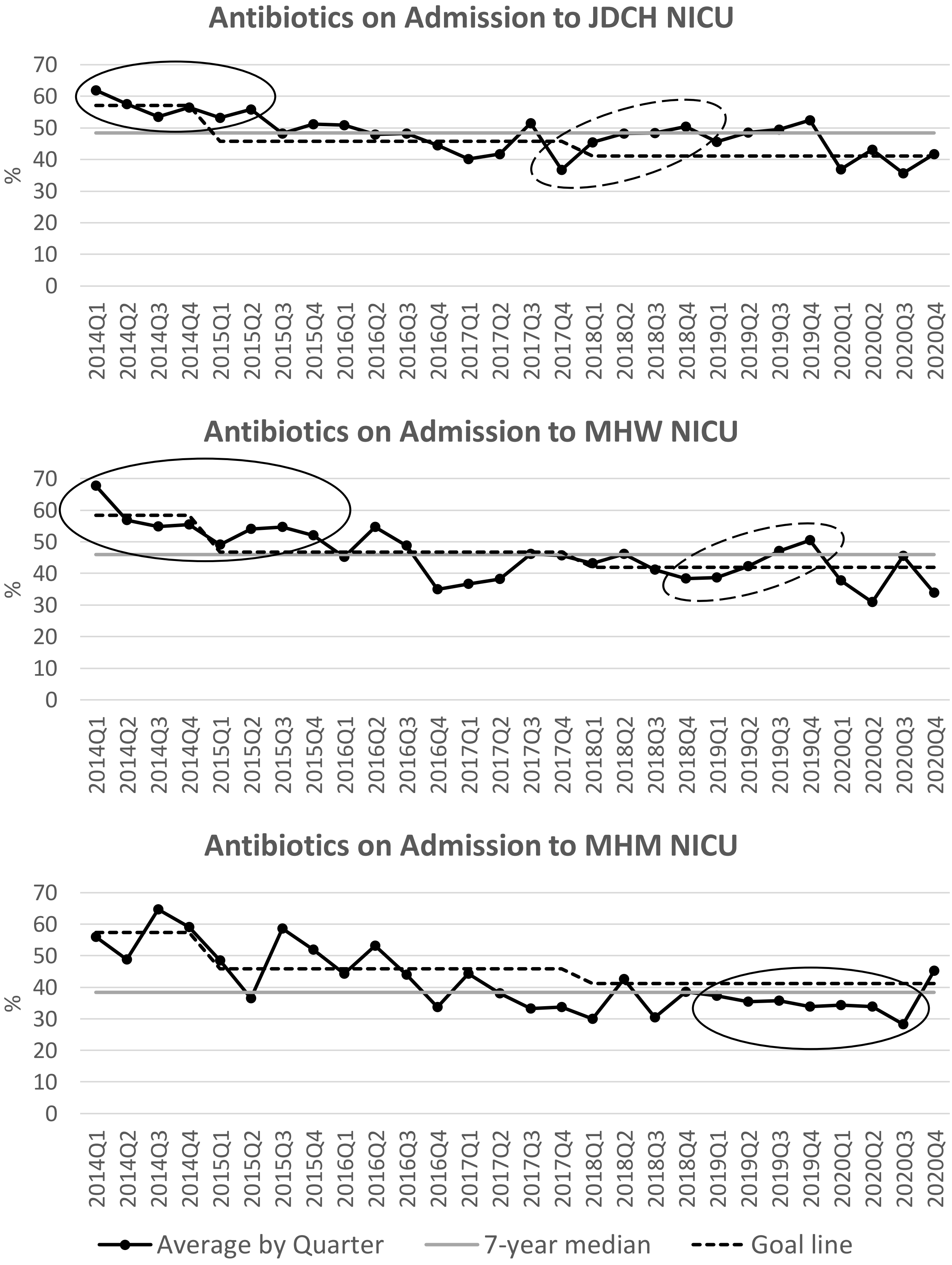

Supplemental Figure 2. Antibiotics continued beyond 72 hours by hospital

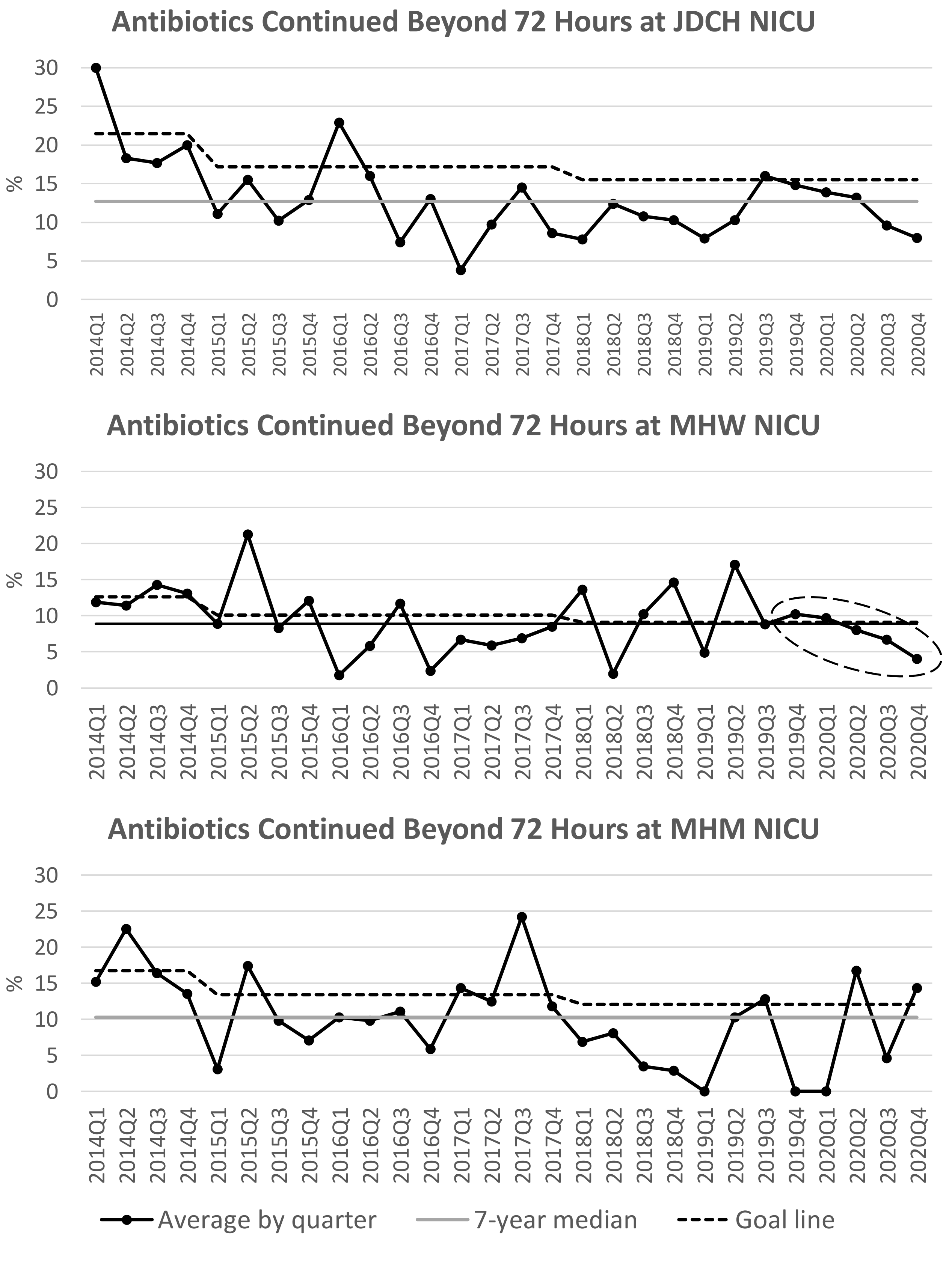

Supplemental Figure 3. Antibiotic utilization rate by hospital

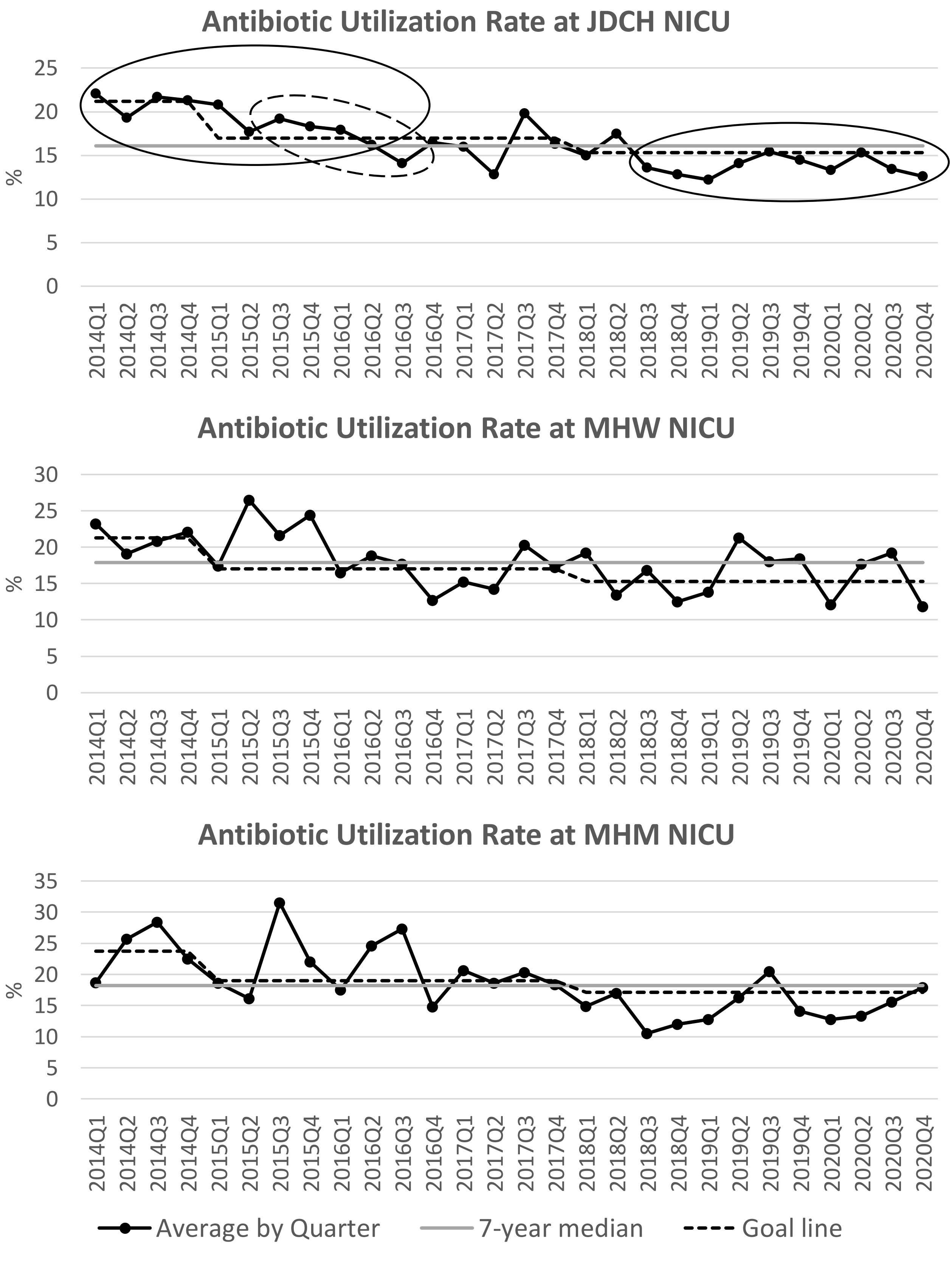

Supplemental figures 1-3. Individual hospital antibiotic stewardship run charts. Goal-line set as baseline period mean for first four quarters (2014), 20% reduction from baseline period for subsequent three years (2015-2017), and an additional 10% reduction for final three years (2018-2020). Seven-year median based on all years of the project including baseline period. Solid and dashed ovals represent shifts (at least six points above or below the median) and trends (at least five points in the same direction), respectively. Other than an adverse trend in antibiotics on admission from 2017Q4 to 2018Q4 at JDCH and 2018Q4 to 2019Q4 at MHW, run charts generally show improvements in shifts and/or trends at each hospital.

Supplemental Digital Content 5

Supplemental Figure 4. Antibiotics on admission by gestational age categories

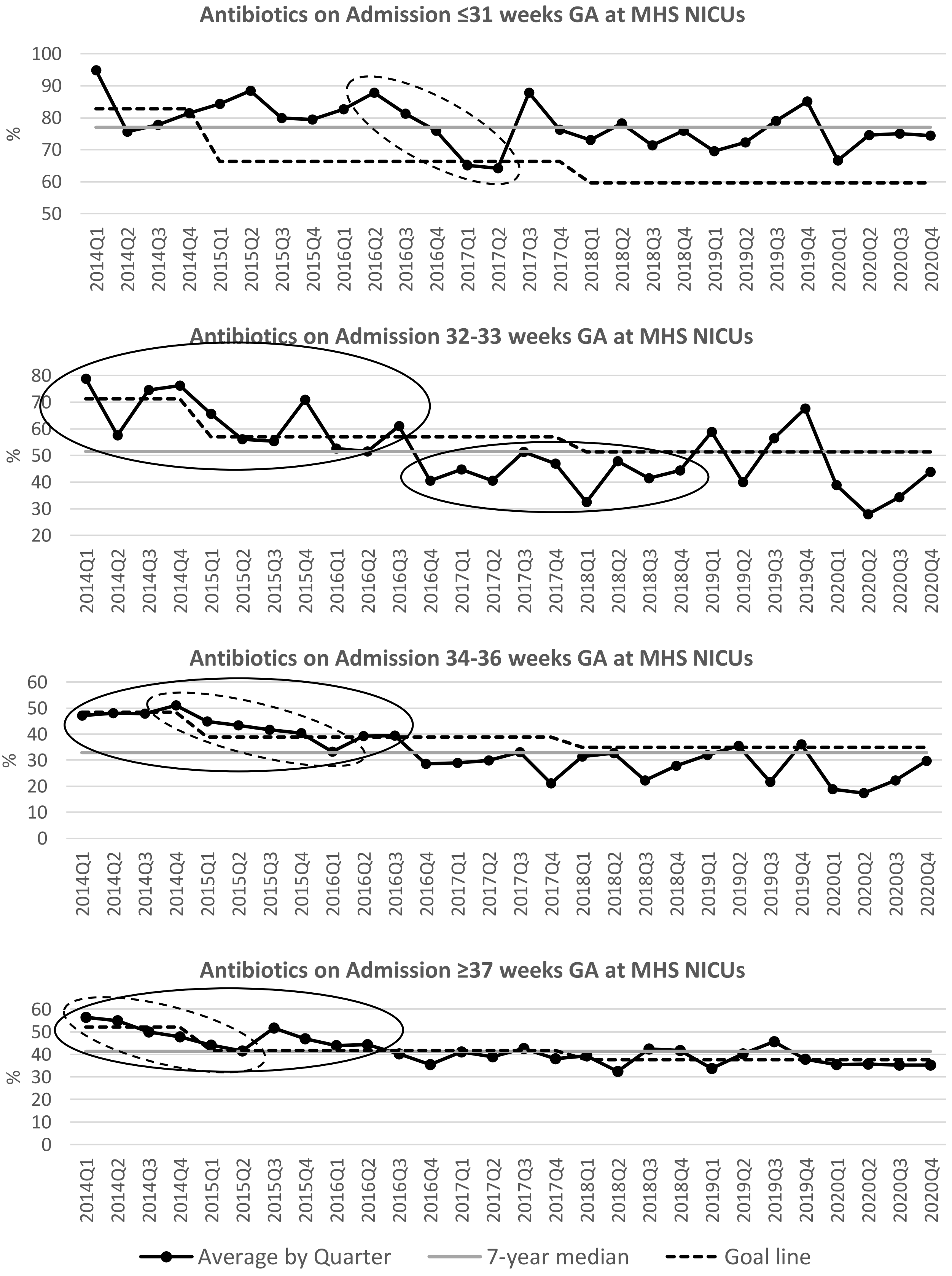

Supplemental Figure 5. Antibiotics continued after 72 hours by gestational age categories

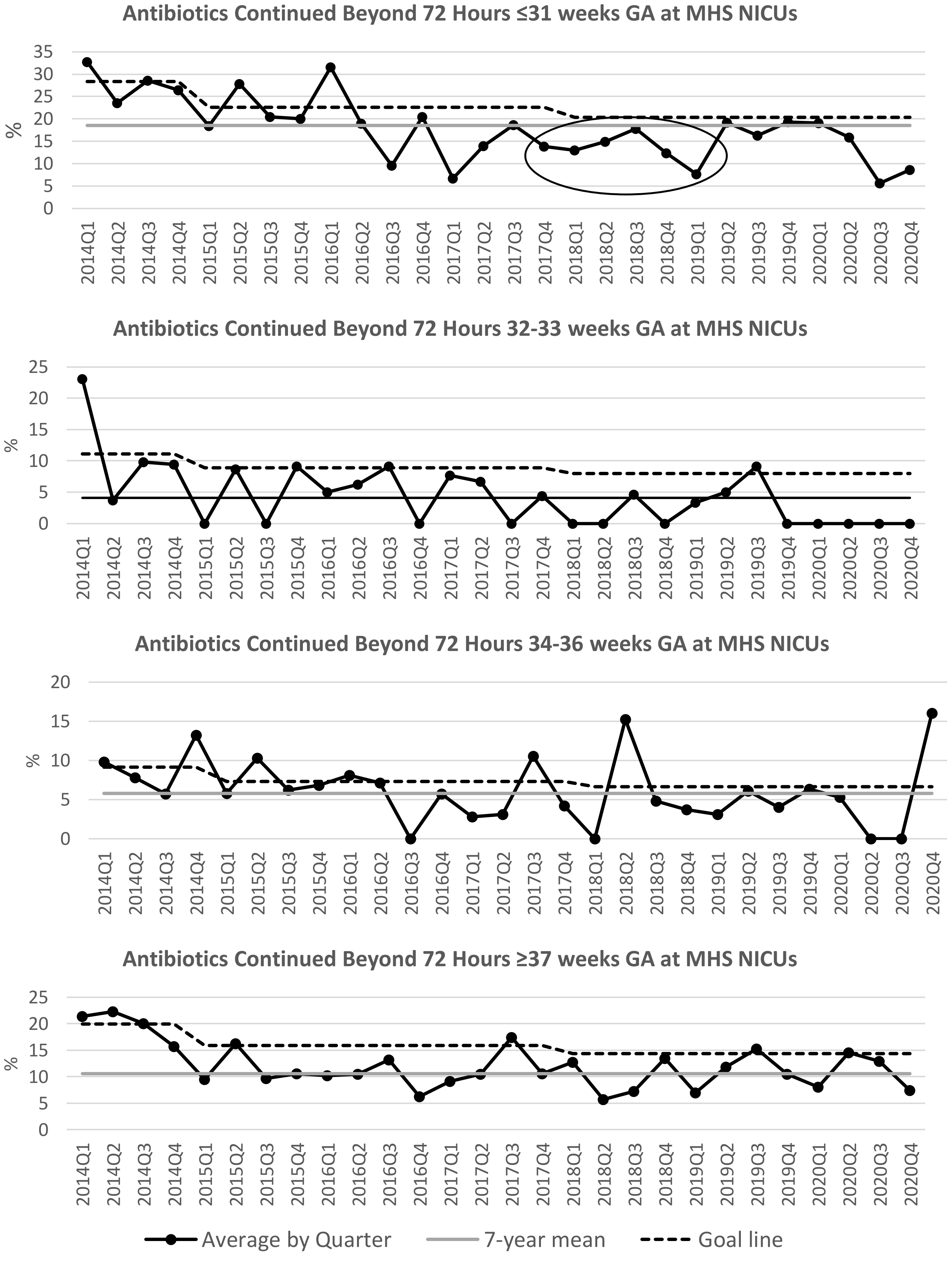

Supplemental Figure 6. Antibiotic utilization rate by gestational age categories

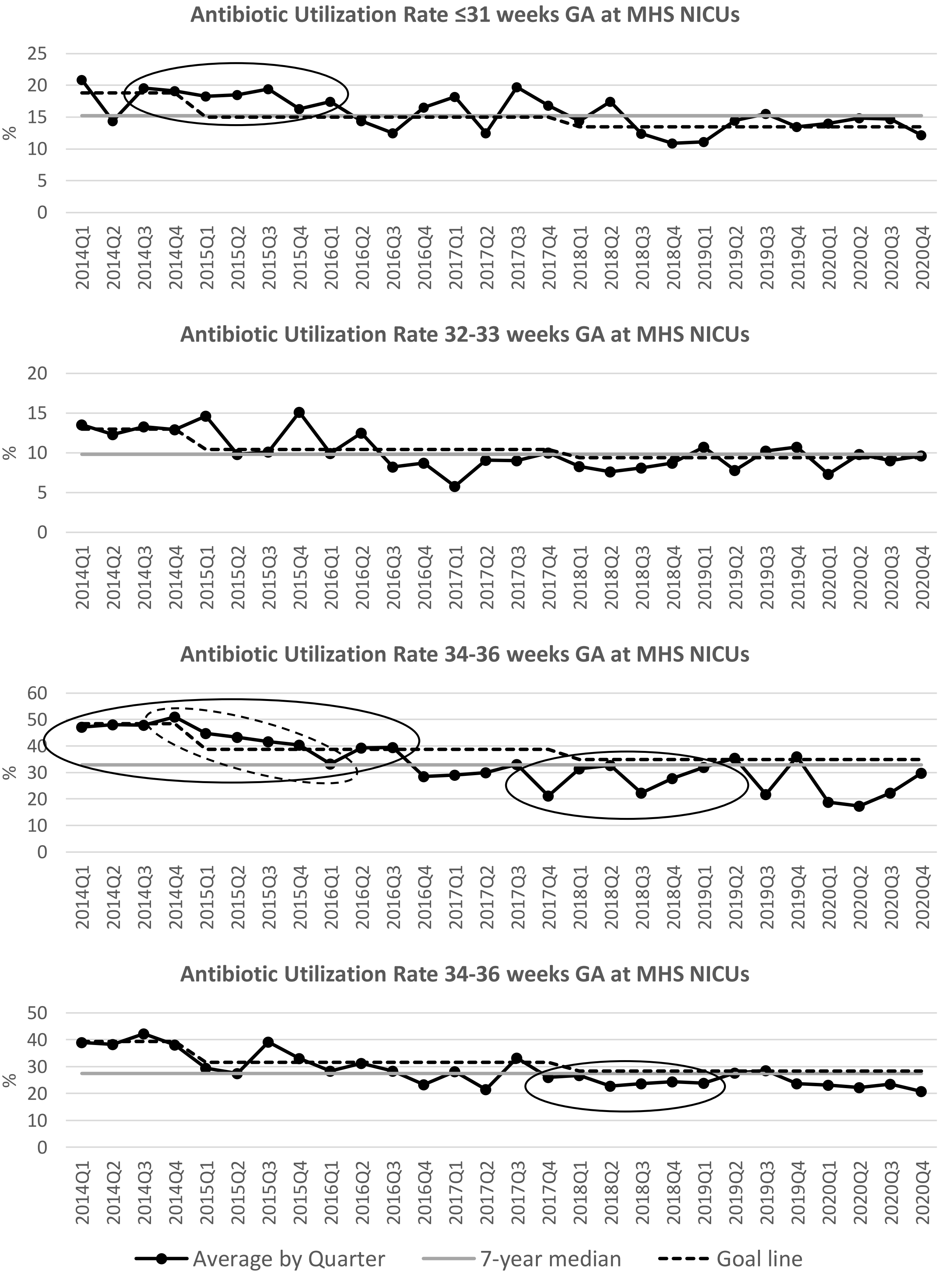

Supplemental figures 4-6. Antibiotic stewardship run charts by gestational age (GA) categories. Goal-line set as baseline period mean for first four quarters (2014), 20% reduction from baseline period for subsequent three years (2015-2017), and an additional 10% reduction for final three years (2018-2020). Seven-year median based on all years of the project including baseline period. Solid and dashed ovals represent shifts (at least six points above or below the median) and trends (at least five points in the same direction), respectively. There were no adverse shifts or trends noted when stratified by GA categories. All GA categories demonstrate shifts and/or trends toward a reduction in antibiotics started on admission, the ≤31 week GA patients a shift toward reduction in antibiotic continuation beyond 72 hours, and all except the 32-34 week patients shifts and/or trends toward reduction in antibiotic utilization rate.

Supplemental Digital Content 6

Supplemental Table 3. Antibiotic reduction goals and results by site*

	2014 (Baseline period)	2019 Goal (↓28% from baseline period)	2020 (Period of sustainability)	Decrease in 2020 compared to 2014
Antibiotics on Admission (AA)				
JDCH	57.1	41.1	39.2	31.3
MHW	58.4	42	37.5	35.8
MHM	57.3	41.3	34.9	39.1
Total (MHS)	57.5	41.4	37.9	34.1
Antibiotics Continued Beyond 72 hours (AC)				
JDCH	21.5	15.5	11.2	47.9
MHW	12.6	9.1	7.1	43.7
MHM	16.7	12	9.4	43.7
Total (MHS)	18.1	13	9.9	45.3
Antibiotic Utilization Rate (AUR)				
JDCH	21.2	15.3	13.7	35.4
MHW	21.3	15.3	15.2	28.6
MHM	23.7	17.1	15	36.7
Total (MHS)	21.5	15.5	14	34.9

* All numbers as percent. 2019 Goal represents combination of two phases of quality improvement initiatives (20% reduction in phase 1 followed by additional 10% reduction in phase 2). 2020 (Period of sustainability) represents actual results during the entire year 2020. Decrease in 2020 compared to 2014 represents the percent reduction in antibiotic usage from 2014 (Baseline period) to 2020 (Period of sustainability). Similar reductions in antibiotic usage was noted at each of our three hospitals and across all three metrics.

JDCH, Joe DiMaggio Children's Hospital; MHS, Memorial Healthcare System; MHM, Memorial Hospital Miramar; MHW, Memorial Hospital West; NICU, neonatal intensive care unit.

Supplemental Table 4. Antibiotic reduction goals and results by gestational age*

Weeks gestational age (GA)	2014 (Baseline period)	2019 Goal (↓28% from baseline period)	2020 (Period of sustainability)	Decrease 2020 compared to 2014
Antibiotics on Admission (AA)				
≤ 31	82.9	59.7	72.4	12.7
32-33	71.2	51.3	36.7	48.5
34-36	48.5	34.9	22.1	54.4
≥ 37	52.1	37.5	35.4	32.1
All GAs	57.5	41.4	37.9	34.1
Antibiotics Continued Beyond 72 hours (AC)				
≤ 31	28.3	20.4	12.7	55.1
32-33	11.1	8	0	100
34-36	9.1	6.6	6.4	29.7
≥ 37	19.9	14.3	11	44.7
All GAs	18.1	13	9.9	45.3
Antibiotic Utilization Rate (AUR)				
≤ 31	18.8	13.5	14	25.5
32-33	13	9.4	8.8	32.3
34-36	15.9	11.4	8	49.7
≥ 37	39.4	28.4	22.5	42.9
All GAs	21.5	15.5	14	34.9

* All numbers as percent. 2019 Goal represents combination of two phases of quality improvement initiatives (20% reduction in phase 1 followed by additional 10% reduction in phase 2). 2020 (Period of sustainability) represents actual results during the entire year 2020. Decrease 2020 compared to 2014 represents the percent reduction in antibiotic usage from 2014 (Baseline period) to 2020 (Period of sustainability). Greater reductions in antibiotic usage were demonstrated for those neonates born 32-33 weeks and 34-36 weeks GA for AA, ≤33 weeks GA for AC, and ≥34 weeks GA for AUR.

GA, gestational age.

Supplemental Table 5. Patients spared antibiotics on admission to the NICU*

	JDCH	MHW	MHM	Total (MHS)
Neonates spared antibiotics on admission, n				
2014	0	0	0	0
2015	47	28	22	97
2016	82	60	50	192
2017	126	60	74	260
2018	77	70	82	229
2019	66	76	72	214
2020	136	70	62	268
Total	534	364	362	1260

* 2014 baseline data was used as reference values to which subsequent years were compared, therefore no patients were spared antibiotics in 2014. Number of patients expected to be started on antibiotics on admission after 2014 was based on percent of patients started on antibiotics in 2014, adjusted for total number of admissions in subsequent years. Patients spared antibiotics on admission was then calculated from each year's expected minus observed values.

JDCH, Joe DiMaggio Children's Hospital; MHS, Memorial Healthcare System; MHM, Memorial Hospital Miramar; MHW, Memorial Hospital West; NICU, neonatal intensive care unit.

Supplemental Table 6. NICU patient days spared antibiotics*

	JDCH	MHW	MHM	Total (MHS)
Patient days spared antibiotics, n				
2014	0	0	0	0
2015	561	-37	36	560
2016	1309	256	129	1694
2017	1067	160	138	1365
2018	1864	277	370	2511
2019	2082	143	223	2448
2020	1844	200	242	2286
Total	8727	999	1138	10864

* 2014 baseline data was used as reference values to which subsequent years were compared, therefore no patient days were spared antibiotics in 2014. Number of antibiotic patient days expected after 2014 was based on percent of antibiotic patient days in 2014, adjusted for total number of patient days in subsequent years. Antibiotic patient days spared was then calculated from each year's expected minus observed values.

JDCH, Joe DiMaggio Children's Hospital; MHS, Memorial Healthcare System; MHM, Memorial Hospital Miramar; MHW, Memorial Hospital West; NICU, neonatal intensive care unit.

Supplemental Table 7. Early onset sepsis cases not initially started on antibiotics[a]

	# true sepsis cases in newborns with delayed antibiotics[b,c], n	# true early onset sepsis cases, n	Incidence delayed antibiotics[b], %	Total MHS NICU admissions	Total MHS births
2015	4	9	44.4	1656	12,357
2016	2	7	28.6	1718	12,373
2017	1	5	20	1600	12,762
2018	3	7	42.9	1670	12,993
2019	1	10	10	1591	12,984
2020	3	9	33.3	1367	11,893
2015-2020 Total	14	47	29.8	9602	75,362

[a] Data from all three NICUs within our healthcare system. MHS early-onset sepsis rate = 0.6 per 1000 live births.

[b] Antibiotics started after 2 hours of life.

[c] Reasons for delayed initiation of antibiotics include late development of signs/symptoms of sepsis (n=8), failure to recognize signs, symptoms, or laboratory values consistent with sepsis (n=4), and asymptomatic until positive blood culture (n=2). Mean (SD) delay in these 14 newborns was 12.6 (7.6) hours. In only one newborn was antibiotics delayed more than 24 hours.

MHS, Memorial Healthcare System; NICU, neonatal intensive care unit.

Supplemental Table 8. 30-day readmissions[a] for bacterial sepsis

	Blood	CSF	Urine	Invasive bacterial infections[b]	Serious bacterial infections[c]	JDCH NICU discharges to home
Pre-antibiotic stewardship						
2010	1	0	0	1	1	701
2011	0	0	0	0	0	679
2012	0	1	0	1	1	824
2013	1	0	0	1	1	779
2014	0	1	0	1	1	913
2010-2014 Total	2	2	0	4	4	3896
Post-antibiotic stewardship						
2015	1	0	2	1	3	970
2016	1	0	3	1	4	976
2017	2	1	1	3	4	825
2018	0	0	3	0	3	761
2019	0	1	1	1	2	714
2020	0	0	0	0	0	671
2015-2020 Total	4	2	10	6	16	4917

[a] Readmission data collected for our level III NICU (JDCH) only, as patients discharged from our community level II NICUs were less likely to be readmitted to our healthcare system.

[b] Invasive bacterial infections = bacteremia or meningitis. Thirty-day readmission rates for invasive bacterial infections pre-antibiotic stewardship (2010-2014) and post-antibiotic stewardship (2015-2020) were 0.1% and 0.12% ($p = 0.8$), respectively.

[c] Serious bacterial infections = bacteremia, meningitis or urinary tract infection. Differences in 30-day readmission rates for serious bacterial infections pre-antibiotic stewardship (2010-2014) and post-antibiotic stewardship (2015-2020) (0.1% vs 0.33%, $p = 0.03$) were primarily attributable to increased number of readmissions for urinary tract infections. 2015-2020 mean (SD) readmission day post-discharge for urinary tract infections was 16 (6.7), indicating an unlikely association between pre-discharge NICU management and post-discharge urinary tract infection. Upon detailed case reviews, no readmissions for serious bacterial infections were deemed preventable or a result of any interventions related to antibiotic stewardship.

CSF, cerebrospinal fluid; JDCH, Joe DiMaggio Children’s Hospital; NICU, neonatal intensive care unit.
